# Effectiveness of a randomized intervention by a geriatric team in frail hospital inpatients in non‐geriatric settings: FRAILCLINIC project

**DOI:** 10.1002/jcsm.13374

**Published:** 2023-11-28

**Authors:** Marta Checa‐López, Alba Costa‐Grille, Alejandro Álvarez‐Bustos, Jose A. Carnicero‐Carreño, Alan Sinclair, Angelo Scuteri, Francesco Landi, Juan José Solano‐Jaurrieta, Srikanth Bellary, Leocadio Rodríguez‐Mañas

**Affiliations:** ^1^ Servicio de Geriatría Hospital Universitario de Getafe Getafe Spain; ^2^ Fundación para la Investigación e Innovación Biosanitaria de Atención Primaria (FIIBAP) Madrid Spain; ^3^ Centro de Investigación Biomédica en Red sobre Fragilidad y Envejecimiento Saludable (CIBERFES) Instituto de Salud Carlos III Madrid Spain; ^4^ Fundación de Investigación Biomédica Hospital Universitario de Getafe Getafe Spain; ^5^ Foundation for Diabetes Research in Older People, Diabetes Frail Medici Medical Practice Luton UK; ^6^ School of Life and Health Sciences Aston University Birmingham UK; ^7^ Department of Clinical and Experimental Medicine University of Sassari Sassari Italy; ^8^ Department of Geriatrics, Neurosciences and Orthopedics Catholic University of the Sacred Heart School of Medicine Rome Italy; ^9^ Instituto de Investigación Sanitaria del Principado de Asturias (ISPA) and Geriatric Service, Monte Naranco Hospital Oviedo Spain; ^10^ Aston University Hospital Birmingham UK

**Keywords:** Frailty, Hospitalization, Intervention, Functional decline, Mortality

## Abstract

**Background:**

Little research has been undertaken on the benefits of frailty management within different hospital settings. The objective of this study is to provide evidence on the viability and effectiveness of frailty management in non‐geriatric hospital settings on mortality and functional decline after discharge.

**Methods:**

Data from the FRAILCLINIC (NCT02643069) study were used. FRAILCLINIC is a randomized controlled trial developed in non‐geriatric hospital inpatient settings (emergency room, cardiology and surgery) from Spain (2), Italy (2) and the United Kingdom (1). Inpatients must met frailty criteria (according to the Frailty Phenotype and/or FRAIL scale), ≥75 years old. The control group (CG) received usual care. The intervention group (IG) received comprehensive geriatric assessment (CGA) and a coordinated intervention consisting in recommendations to the treating physician about polypharmacy, delirium, falls, nutrition and physical exercise plus a discharge plan. The main outcomes included functional decline (worsening ≥5 points in Barthel Index) and mortality at 3 months. We used multivariate logistic regression models adjusted by age, gender and the Charlson index. Intention‐to‐treat (ITT) and per‐protocol (PP) analyses were used.

**Results:**

Eight hundred twenty one participants (IG: 416; mean age 83.00 ± 4.91; 51.44% women; CG: 405; mean age 82.46 ± 6.03; 52.35% women) were included. In the IG, 77.16% of the participants followed the geriatric team's recommendations as implemented by the treating physicians. The intervention showed a benefit on functional decline and mortality [OR: 0.67(0.47–0.96), *P*‐value 0.027 and 0.29(0.14–0.57), *P*‐value < 0.001, respectively) when fully followed by the treating physician. A trend to benefit (close to statistical significance) in functional decline and mortality were also observed when any of the recommendations were not followed [OR (95% CI): 0.72 (0.51–1.01), *P*‐value: 0.055; and 0.64 (0.37–1.10), *P*‐value: 0.105, respectively].

**Conclusions:**

An individualized intervention in frail in‐patients reduces the risk of functional deterioration and mortality at 3 months of follow‐up when a care management plan is designed and followed.

## Background

Population ageing and the concomitant rise in the number of frail older adults represent a serious health and social care challenge.^1^ Older people are frequent users of hospital care,^2^ especially those with physically debilitating geriatric syndromes like frailty,^3^ increasing the pressure on healthcare systems worldwide.^1^

Frailty is a geriatric syndrome characterized by a diminished capacity to respond to stressors, as a result of a reduced functional reserve.^4^ This condition is not only related to poorer health outcomes,^5^ but also to increased health care costs and service use.^6^ Frailty and hospitalization are frequently associated with each other.

Frailty is highly prevalent among the inpatient hospital setting varying from 27% to 80%.^6^, ^7^ Acute hospitalization is a major contributor to functional decline and disability in frail older adult.^7^, ^8^ Due to this, establishing strategies to detect and treat frailty with the intention to foster a longer and healthier life during hospitalization have been proposed.^9^

Some evidence has demonstrated the benefits of acute care for the elderly (ACE) units in terms of functional decline and institutionalization reduction in a mid‐term.^10^, ^11^ However, an important percentage of frail older patients admitted to the hospital is not managed in ACEs due to their high occupation rates or the lack of ACEs in some hospitals, excluding them from the potential advantages of global ACES management focused on the detection and integrated approach carried to frailty.^10^, ^11^ These undetected frail patients are at a special risk of adverse outcomes.^12^ Nevertheless, evidence of the interventions' effectiveness in frail older adults admitted to the hospital out of the ACEs is lacking.^13^, ^14^, ^15^ Previous research has found that comprehensive geriatric assessment (CGA) and management has a strong beneficial effect on general older in‐patients, not only in ACEs but also in other hospital settings.^16^ However, the evidence about these benefits in frail older people is lacking. A recent systematic review about interventions on frailty in hospitalized frail older adults states that due to the low number of RCTs carried out in hospital setting and the low quality of the existing studies,^17^ there is need for new RCTs to be carried out to generate a protocol appropriate for frail older people. Moreover, most of the studies are focused on mortality while studies assessing functional outcomes are of special interest in this older population.^16^

This study aims to evaluate the effectiveness of the detection of frailty and intervention on it by a geriatric consultation team in inpatients at non‐geriatric in‐hospital settings,^17^ to prevent mortality, functional decline (progression of frailty status and disability) and other adverse events (institutionalization, hospital readmission and recurrent emergency visits) after discharge.

## Methods

### Trial design

FRAILCLINIC (NCT02643069) was a phase III multicentric randomized clinical trial conducted between June 2016 and March 2021. We recruited inpatients older than 75 years admitted to some of the following clinical settings: emergency room (ER), cardiology and surgery (both as an elective‐ES and urgent surgery‐US). The study was carried out in two hospitals in Spain (University Hospital of Getafe and Monte Naranco Hospital, Oviedo), two in Italy (Sacro Cuore University Hospital and San Rafael Hospital, both in Rome) and one setting in the United Kingdom (Luton & Dunstable University Hospital, that was substituted by the Aston University Hospital in the last 6 months of the trial). The settings were selected based on the availability of the centers where the study was conducted. Oncology department was also initially included, but was finally discarded in the early phases of the study due to lack of recruitment. Participants were recruited consecutively among all the patients (age > 75 years) who attended the four settings of the study. The setting of care registered in the database for every participant was the one where they were contacted for the first time by the Geriatric Team. Researchers checked every day those admissions and excluded those with data informing about exclusion criteria in the electronic clinical records. After this first selection, they met the potential participants and after obtaining their verbal acceptance to be screened, they were assessed regarding their frailty status at admission by means of two validated frailty tools: the Fried's Frailty Phenotype (FP)^4^ and the FRAIL scale.^18^

Very briefly, the Fried Frailty Scale assesses five domains: fatigue, involuntary weight loss, slow gait speed, low grip strength and low levels of physical activity. The FRAIL scale evaluates fatigue, resistance, ambulation, illnesses and loss of weight. Using either tool, an individual is frail if they met three or more of the five frailty criteria. Exclusion criteria were moderate to severe cognitive impairment (Mini‐Mental State Examination (MMSE)^19^ ≤ 18), severe functional impairment (Barthel Index^20^ < 40), life expectancy <6 months, predicted by their treating physician according to their clinical situation, ICU admission in the first 24 h of admission or any other clinical condition that could interfere in the correct understanding of the project or preventing informed consent. All the patients enrolled in the study signed an informed consent.

Baseline characteristics for both groups were recorded during the first 24 h after admission, including demographic and anthropometric characteristics, medical history (admission diagnosis and co‐morbidities) and medication use recorded from clinical records. Basal (1 week before admission) functional [Barthel Index (which ranges from 0 to 100, the latter the best independence score) and Lawton scale (scoring from 0 to 6, being 6 the score for an independent status^21^)] and cognitive status [MMSE (which ranges from 0 to 30, being 30 the highest cognitive status)] at admission were recorded. Finally, as previously mentioned, frailty was also assessed at admission through two different tools: the Fried's Frailty Phenotype^4^ and the FRAIL scale.^18^

Participants were randomized 1:1 automatically using a random number registry conducted by a blinded manager at each centre.

### Description of the intervention and control groups

*Intervention group (IG)*: Patients assigned to the IG received, in addition to the usual care provided in the hospital setting where they were admitted according to their existing local protocols and guidelines, a comprehensive geriatric assessment, including a functional, cognitive, mood, nutritional and social assessment, designed and followed by a plan addressed by a geriatric team, covering both in‐hospital and post discharge time. The recommendations during hospitalization embrace the following five aspects: polypharmacy, delirium, falls, nutrition and physical exercise, adapted to the needs of each patient. These recommendations could be refined during the hospital stay and extended after discharge, depending upon the progress of the patient during the hospitalization period. In addition, the intervention included a coordination with primary and social care, rehabilitation and discharge plan, in order to establish a multidisciplinary care strategy to optimize resources and achieve the highest degree of independence and quality of life for the patient. Taking into account the diversity of clinical models of practice, the way of providing the interventions both during hospitalization and after discharge were modified according to local practice in each of the participant hospitals.

The geriatric team provided the recommendations for each patient to the treating physician by two concurrent sources: writing them in the clinical record plus explaining to him/her in person, as they were responsible for implementation of the intervention. The team was composed of by a ‘core’ team (geriatrician and nurse) plus any other professional (physiotherapist, sport science professionals, nutritionist, registered dietitians, pharmacist, occupational therapist, psychologist and social worker) needed to improve the assessment or the design of the interventions in any particular patient. Patients were visited daily during their stay to check if the recommended measures had been implanted, updating them according to the clinical evolution, and reminding the treating physician if they had not been implemented. Only those patients where all the recommendations were implemented by his or her treating physician were considered ‘compliant’.

*Control group (CG)*: Patients allocated into the CG received the usual care provided in each one of the hospital settings where the participant had been admitted, according to their existing local protocols and guidelines. This usual care could include consultation on demand with the geriatric department by the patient's treating physician.

Both groups underwent telephone follow‐up at 3 months ± 1 week after hospital discharge. The researchers who carried out this follow‐up were blinded regarding the patient's group, intervention or control.

### Adverse events

Information about the following adverse events were collected: constipation, urinary incontinence, instability, falls, adverse drugs reactions, hypoglycaemia, low blood pressure (<100 mmHg of systolic blood pressure), delirium and nosocomial infections.

## Outcomes

The primary outcomes are the effects of the intervention on functional decline (assessed by the Barthel Index), institutionalization (yes/no) and mortality (yes/no) 3 months after discharge. With reference to functional decline, a worsening in Barthel score (≥5 points) regarding the basal (pre‐hospitalization) score, was considered the event. Secondary outcomes included disability in the instrumental daily life activities (assessed by the Lawton scale), visits to the ER (yes/no), hospital readmissions (yes/no) and changes in the frailty status, evaluated by the FRAIL scale. Worsening in the Lawton scale score (≥1 point) and improvement in the FRAIL scale score (≥1 item) were considered the event for the analysis.

Baseline visits were performed face to face. Follow‐up visits after discharge (at 3 months ± 1 week) were carried out by telephone. In this follow‐up phone call, all the outcomes were assessed.

## Statistical analysis

We calculated the sample size to minimize the estimation bias. For doing so, it is recommended to have a minimum of five and seven outcome events per independent variable (intervention, age, gender and Charlson Index) (Peduzzi et al., J Clin Epidemiol 1996). This means that for a 95% confidence interval (alpha error: 0.05), we need a minimum number of 28 events. Based on these considerations, sample size was calculated using the formula

$$n^{*}p\pm z_{1-\frac{\alpha }{2}}\sqrt{n^{*}p^{*}\left(1-p\right)}=28,$$

where *P*‐value is 0.3 (30% of functional impairment in older people hospitalized (Hoogendijk 2013, Umegaki et al., Eur Geriatr Med 2022) and ‘n’ is the sample size to be calculated. The calculated sample size was 85, for each hospital and group. This means a total sample size of 680 individuals. Moreover, a loss rate of 15% were assumed, so that the final sample size was 782 individuals (156 participants per hospital). It means 156 participants per hospital.

Descriptive data were shown as mean (standard deviation) and frequency (%) for continuous or categorical variables, respectively. The comparisons between groups were assessed using Mann–Whitney test and *χ* ^2^ test for continuous or categorical variables, respectively.

We used multivariate logistic models to assess the effect of the intervention on adverse outcomes. We used age, gender and Charlson Index^22^) as potential confounders. Taking into account the rate of treating physicians who did not follow any of the recommendations of the geriatric team, two types of statistical analyses were conducted: one according to the intention‐to‐treat principle (ITT) and another using the as‐treated or per‐protocol (PP). As we do not know which component(s) of the intervention accounts for the benefit, we separated the participants in the IG into two categories: ‘compliant’ those participants whose treating physician followed all the recommendations (PP analysis) and ‘non‐compliant’, those participants who did not follow one or more of the recommendations (who were included, jointly with those following the recommendations, for the ITT analysis). No differences were found either in terms of age (*P*‐value 0.68), gender (*P*‐value 0.10), Charlson Index score (*P*‐value 0.15), Barthel Index (*P*‐value 0.15), Lawton scale (*P*‐value 0.38), MMSE score (*P*‐value 0.09), and frailty according to the Frailty Phenotype (*P*‐value 0.71) or the FRAIL score (*P*‐value 0.93) between the participants included in the PP analysis versus those in the ITT analysis. In addition, we performed two sensitivity analyses in case the intervention was particularly effective in any of the settings in which the intervention was performed, or in patients who were frail according to any of the scales used to assess frailty.

The significance level was established at *P*‐value <0.05. Statistical analyses were performed with the Statistical Package R Version 3.6.1 for Windows (Vienna, Austria).

## Results

**Figure 1:**
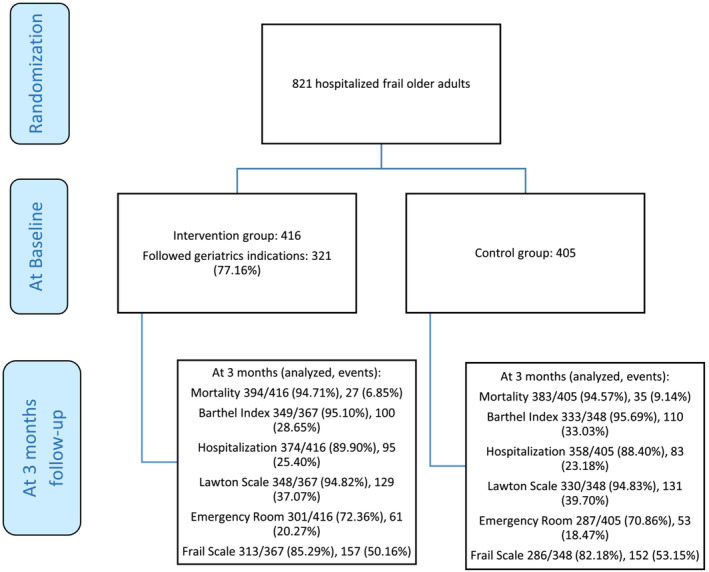
Study flow chart diagram.

A total of 821 subjects were recruited with 405 being randomly assigned to the control group and 416 to the intervention group (Figure 1). The mean age was 82.46 (6.03 SD) years for the intervention group and 83.00 (4.91 SD) years for the control group. 51.44% in the intervention group and 52.35% in the control group were women (Table 1). Assessment of the main outcomes was carried out in 94.71% (mortality) and 95.1% (functional decline) in the IG and 94.57% and 95.69%, respectively, in the CG (Figure 1). Among IG and CG participants, 89.7% and 89.4%, respectively, were classified as frail according to the Frailty Phenotype, and 76.2% and 75%, according to FRAIL scale. Participant characteristics in the different clinical settings are shown in Table S1.

**Table 1:** Clinical and functional values by treatment group.

Variable	Int	Con	*P*‐value
*N*	416	405	
Age (years)	83.00 (4.91)	82.46 (6.03)	0.261
Gender (% female)	214 (51.44)	212 (52.35)	0.796
Barthel score, mean (SD)	83.94 (13.38)	84.83 (12.98)	0.370
Lawton score, mean (SD)	2.27 (1.07)	2.35 (1.02)	0.190
Charlson score, mean (SD)	6.95 (2.19)	6.56 (2.12)	**0.019**
Frailty Phenotype (% Frail)	373 (89.66)	362 (89.38)	0.767
FRAIL scale (% Frail)	317 (77.13)	304 (76.00)	0.992
MMSE score, mean (SD)	24.42 (3.37)	24.49 (3.40)	0.710
Length of stay, mean (SD)	13.01 (11.15)	12.10 (11.36)	**0.204**

*Note*: In bold: *P*‐value <0.05. Values for age and all scores are shown in mean and standard deviation.

Abbreviation: MMSE, Mini‐Mental State Examination.

CAG of the participants was followed by the building of a care plan in the IG in all cases, although this was not the case for the implementation of the recommendations by the treating physician. In fact, 321 subjects within the intervention group complied with the recommendations given by the geriatric team, which represented 77.16% of that group sample, while the treating physician did not implement the care plan designed by the team in 95 (22.84%) participants.

Regarding the primary outcome, the intervention showed a decrease in the risk of worsening the Barthel Index at 3 months after discharge ranging from 28% [OR (95% CI) 0.72 (0.51–1.01; *P*‐value 0.055)] to 33% [OR: 0.67 (0.47–0.96; *P*‐value 0.027)] depending upon the type of analysis run (Intention to Treat or Per Protocol, respectively). When we looked at the effect in the different settings of care, we found statistically significant differences in the ER, both in the ITT and per protocol analysis, 0.49 (0.26–0.91; *P*‐value 0.024) and 0.53 (0.28–0.99; *P*‐value 0.047), respectively. Concerning to mortality, there was a lower risk of dying within 3 months for patients who complied with the recommendation of geriatricians [OR (95% CI) = 0.29 (0.14–0.57); *P*‐value <0.001]. The same result was shown in the ER [OR (95% CI) = 0.31 (0.10–1.00; *P*‐value 0.049)] and cardiology settings (OR 0.21 (0.04–0.99; *P*‐value 0.049)) in the per protocol analysis. Alternatively, only 20 subjects in both groups (9 in the intervention group and 11 in the control group) were institutionalized at 3 months, and we could not find statistically significant differences in this outcome (Table 2).

**Table 2:** Effect of intervention on the primary outcomes (odds of worsening of the Barthel index score and mortality) at 3 months per setting according to the intention‐to‐treat and per protocol analysis.

	Intention to treat analysis		Per protocol analysis	
	OR (95% CI)	*P*‐value	OR (95% CI)	*P*‐value
Barthel Index				
All	0.72 (0.51–1.01)	0.055	0.67 (0.47–0.96)	**0.027**
ER	0.49 (0.26–0.91)	**0.024**	0.53 (0.28–0.99)	**0.047**
Car	0.94 (0.46–1.93)	0.876	0.88 (0.42–1.84)	0.742
ES	0.61 (0.28–1.34)	0.220	0.45 (0.19–1.05)	0.065
US	0.98 (0.48–2.00)	0.947	0.96 (0.45–2.03)	0.915
Mortality				
All	0.64 (0.37–1.10)	0.105	0.29 (0.14–0.57)	**<0.001**
ER	0.65 (0.25–1.72)	0.390	0.31 (0.10–1.00)	**0.049**
Car	0.60 (0.20–1.82)	0.366	0.21 (0.04–0.99)	**0.049**
ES	0.70 (0.15–3.27)	0.654	0.45 (0.07–2.84)	0.393
US	0.52 (0.19–1.46)	0.216	0.28 (0.07–1.07)	0.062

*Note*: In bold: *P*‐value <0.05.

Abbreviations: Car, cardiology; CI, confidence interval; ER, emergency room; ES, elective surgery; OR, odds ratio; US, urgent surgery.

According to the secondary outcomes (Table 3), our intervention had benefit in the probability of worsening the Lawton scale at 3 months of discharge, OR 0.71 (0.50–1.00; *P*‐value 0.047), in those who received geriatric team intervention. No other statistically significant differences were found in the whole sample in relation with the secondary outcomes. When we analysed differences by settings, in the bivariant analysis, we found a better Lawton score in the intervention group in the patients attended in the ER (2.70 ± 1.10 vs. 2.36 ± 1.06; *P* = 0.022) while the opposite was shown in urgent surgery (1.91 ± 0.82 vs. 2.41 ± 0.92; *P* = 0.001). Length of stay was lower in the intervention group in elective surgery (9.14 ± 6.20 days vs. 10.54 ± 7.72; *P* = 0.042) and there was also a minor benefit in the percentage of patients showing a worsening in the Barthel Index in the intervention group (29.79% vs. 44.09%; *P* = 0.042) in participants attended in the ER (Table S1). Nevertheless, in the adjusted analysis, we only found benefits in the cardiology setting: geriatric team intervention reduced readmissions at 3 months, [OR (95% CI) = 0.45 (0.22–0.89); *P*‐value 0.023], and improved frailty, measured by the FRAIL scale [OR (95% CI) = 2.00 (1.04–3.83); *P*‐value 0.038] in the ‘per protocol’ analysis.

**Table 3:** Effect of intervention on the secondary outcomes (odds of worsening of the Lawton index and FRAIL scale, re‐admissions and visits to the emergency room) at 3 months per setting according to the intention‐to‐treat and per protocol analysis.

	Intention to treat analysis		Per protocol analysis	
	OR (95% CI)	*P*‐value	OR (95% CI)	*P*‐value
Re‐admission				
**All**	1.01 (0.71–1.44)	0.949	0.83 (0.57–1.19)	0.308
ER	1.58 (0.82–3.06)	0.170	1.02 (0.52–1.99)	0.958
Car	0.45 (0.22–0.89)	**0.023**	0.43 (0.2**0**–0.89)	**0.023**
ES	1.47 (0.60–3.59)	0.397	1.37 (0.55–3.43)	0.504
US	1.23 (0.60–2.54)	0.576	0.99 (0.47–2.09)	0.985
Lawton scale				
**All**	0.91 (0.66–1.26)	0.566	0.71 (0.50–1.00)	**0.047**
ER	0.85 (0.44–1.62)	0.619	0.94 (0.49–1.82)	0.852
Car	0.66 (0.35–1.24)	0.195	0.69 (0.36–1.32)	0.262
ES	0.64 (0.31–1.32)	0.229	0.27 (0.12–0.62)	**0.002**
US	1.67 (0.82–3.38)	0.156	1.31 (0.63–2.74)	0.470
Visits to emergency room				
**All**	1.01 (0.66–1.54)	0.980	0.8 (0.51–1.25)	0.323
ER	1.52 (0.70–3.30)	0.286	1.2 (0.55–2.63)	0.640
Car	1.38 (0.51–3.74)	0.523	1.09 (0.4**0**–3.02)	0.861
ES	0.84 (0.32–2.22)	0.723	0.64 (0.22–1.89)	0.423
US	0.52 (0.22–1.23)	0.134	0.42 (0.16–1.1**0**)	0.079
FRAIL scale				
**All**	0.9 (0.64–1.25)	0.530	0.95 (0.67–1.33)	0.754
ER	0.81 (0.43–1.52)	0.513	0.82 (0.43–1.55)	0.533
Car	2.00 (1.04–3.83)	**0.038**	1.95 (1.01–3.76)	**0.048**
ES	0.48 (0.22–1.03)	0.060	0.69 (0.31–1.54)	0.365
US	0.53 (0.25–1.15)	0.109	0.52 (0.23–1.14)	0.102

*Note*: In bold: *P*‐value <0.05.

Abbreviations: Car, cardiology; CI, confidence interval; ER, emergency room; ES, elective surgery; OR, odds ratio; US, urgent surgery.

After this initial analysis, we separated our sample in order to explore the effect of the intervention on the participants depending upon the frailty tool which identified them as frail at baseline [(Frailty Phenotype or the FRAIL scale (Tables 4 and S2) or both (Table S3)]. Those participants who were frail according to the FP, regardless of their frailty status according to the FRAIL scale, had a significantly lower risk of worsening in the Barthel Index [OR (95% CI) = 0.66 (0.46–0.94); *P*‐value 0.021] (Table 4). By opposite, this effect was not shown in the participants qualified as frail according to the FRAIL scale. Nevertheless, in terms of mortality risk reduction, the benefit of the intervention is shown regardless of the frailty tool used [OR (95% CI) = 0.28 (0.14–0.59); *P*‐value 0.001 and OR (95% CI) = 0.36 (0.18–0.74); *P*‐value 0.005, according to FP and FRAIL scale, respectively] (Table 4) and in those who were frail according to both tools in the per protocol analysis [HR (95% CI) = 0.38 (0.18; 0.80); *P*‐value 0.011] (Table S3). Regarding the worsening in the Lawton scale, we found a protective effect of the intervention in those who were frail according to the FRAIL scale [OR (95% CI) = 0.66 (0.46–0.96); *P*‐value 0.031] and in those frail subjects according to both tools [OR (95% CI) = 0.67 (0.45–0.99); *P*‐value 0.046] (Tables S2 and S3).

**Table 4:** Intervention effect on the primary outcomes (odds of worsening of the Barthel index score and mortality) selecting those participants who were frail exclusively according to the frailty phenotype or the FRAIL scale.

	Frail according to the Frailty Phenotype				Frail according to the FRAIL scale			
	Intention to treat analysis		Per protocol analysis		Intention to treat analysis		Per protocol analysis	
	OR (95% CI)	*P*‐value	OR (95% CI)	*P*‐value	OR (95% CI)	*P*‐value	OR (95% CI)	*P*‐value
Barthel Index								
**All**	0.66 (0.46; 0.94)	**0.021**	0.62 (0.43; 0.90)	**0.011**	0.76 (0.52; 1.12)	0.160	0.82 (0.55; 1.23)	0.342
ER	0.52 (0.27; 0.98)	**0.044**	0.54 (0.28; 1.04)	0.065	0.54 (0.27; 1.07)	0.076	0.67 (0.33; 1.33)	0.249
Car	0.73 (0.33; 1.62)	0.432	0.67 (0.30; 1.53)	0.347	0.81 (0.38; 1.74)	0.588	0.84 (0.39; 1.85)	0.673
ES	0.52 (0.24; 1.15)	0.109	0.39 (0.17; 0.94)	**0.035**	0.82 (0.33; 2.02)	0.666	0.69 (0.26; 1.81)	0.445
US	0.98 (0.45; 2.13)	0.962	0.52 (0.24; 1.15)	0.109	2.29 (0.79; 6.64)	0.126	0.82 (0.33; 2.02)	0.666
Mortality								
**All**	0.66 (0.37; 1.17)	0.154	0.28 (0.14; 0.59)	**0.001**	0.75 (0.41; 1.36)	0.341	0.36 (0.18; 0.74)	**0.005**
ER	0.66 (0.25; 1.75)	0.407	0.32 (0.10; 1.01)	0.052	0.65 (0.25; 1.73)	0.390	0.32 (0.10; 1.01)	0.052
Car	0.66 (0.15; 2.83)	0.574	0.17 (0.02; 1.56)	0.118	0.45 (0.14; 1.46)	0.182	0.19 (0.04; 0.91)	**0.037**
ES	0.64 (0.14; 2.99)	0.574	0.42 (0.07; 2.68)	0.360	1.82 (0.28; 11.64)	0.528	0.98 (0.12; 7.99)	0.988
US	0.29 (0.07; 1.12)	0.072	0.64 (0.14; 2.99)	0.574	0.69 (0.15; 3.06)	0.623	1.82 (0.28; 11.64)	0.528

*Note*: In bold: *P*‐value <0.05. Models adjusted by age, gender and Charlson Index.

Abbreviations: Car, cardiology; CI, confidence interval; ER, emergency room; ES, elective surgery; OR, odds ratio; US, urgent surgery.

Additionally, we assessed the number needed to treat (NNT) to measure the effect of the intervention in those variables in which we found that the intervention had an impact. The estimated NNT for mortality was 44. In relation to the improvement in the Barthel and Lawton scales compared with control patients, the NNT were 19 and 13, respectively. Furthermore, the NNT to avoid a poor outcome in those same outcomes in IG than in the CG were 23 and 38, respectively.

We detected 547 adverse events, 225 in 152 participants of the UCG and 322 in 209 participants of the IG. None of these adverse events were related to the intervention, nor motivated the exclusion of the study. Except for constipation (115/405 in UCG vs. 165/416 in IG; *P*‐value <0.001), there were no significant differences among groups in the adverse events: urinary incontinence (48/405 in UCG vs. 69/416 in IG, *P*‐value 0.054), instability (12/405 in UCG vs. 12/416 in IG; *P*‐value 0.947), falls (8/405 in UCG vs. 7/416 in IG; *P*‐value 0.754), adverse drugs reactions (13/405 in UCG vs. 21/416 in IG; *P*‐value 0.186), low blood pressure (3/405 in UCG vs. 9/416 in IG; *P*‐value 0.0895), delirium (0/405 in UCG vs. 1/416 in IG; *P*‐value NA) and nosocomial infections (26/405 in UCG vs. 33/416 in IG; *P*‐value 0.401).

## Discussion

The benefits of interventions on frail hospital inpatients are far to be proven.^13^, ^14^, ^15^ Our results demonstrate that, in these frail inpatients, CGA along with an individualized care plan designed and monitored by a geriatric team in high‐risk settings, is a protective factor for worsening of the functional status (assessed by the Barthel Index score and the Lawton scale), and for mortality at 3 months.

This effect was observed when the treating physician followed the recommendations of the geriatric team. It is worthy to mention that, taking into account previous reports showing a low rate of compliant by the treating physician of the recommendations of a geriatric team,^23^ part of our intervention consisted in the robust follow‐up of the patients and the frequent contact with the treating physician, in an effort to have our recommendations fully implemented. The aim was achieved in near 80% of the cases. However, we ran two different analyses (ITT and PP) trying to assess the relevance of the fully compliant of the geriatric team recommendations. Our results suggest that indeed with these high rates of compliant, there is still opportunity for improvement, mainly in the case of mortality, where ORs were much lower in the PP analysis, while the difference was quite marginal in the case of functional decline. Furthermore, it is important to highlight the power of our results seen on the NNTs related to the outcomes, mainly the functional ones. Finally, the intervention seems to be quite safe. Adverse events were not significantly different between groups except for constipation, a usual but not life‐threatening symptom in older people.

A Cochrane systematic review showed that the use of CGA in older adults admitted to hospital increases the likelihood of being ‘alive and in their own homes’ from 6 weeks to 12 months of follow‐up but did not find differences when mortality was treated as the only variable in the same period of follow‐up.^15^ This study did not find any effect of the CGA in terms of dependence or re‐admission. Nevertheless, this systematic review and meta‐analysis was not only performed in frail participants, including only five studies in which frailty was a criterion for targeting delivery of CGA.^15^

There is a high disparity between tools in the identification of frail patients and this fluctuates between the settings of care^24^ with different risks of adverse events associated with each one.^25^ On the other hand, an umbrella review of systematic reviews showed that CGA in the hospital medical settings, reduced nursing home admission, the risk of falls and pressure sores in generally older people in medical departments as oncology, haematology and ER.^16^ This same review showed that CGA intervention decreases the risk of developing physical frailty in community dwelling non‐frail older people.

In our study, the greatest benefit is shown in the ER and in Cardiology. However, the aim of the study was not to assess differences among settings and because of that the sample size (and accordingly the power to detect differences) was calculated for the whole sample. As a consequence, there is a potential for additional differences that did not reach statistical significance in our analysis due to the above mentioned reason. Moreover, the marginal statistical significance of these findings in our study makes them less reliable and, indeed, merits further confirmation in future studies.

Hospitalization is one of the most stressful events for older adults,^17^, ^26^ being one of the main factors of catastrophic disability in this population.^26^ Disability is the end‐stage in the functional continuum, which is usually preceded by frailty. The effectiveness of interventions in hospitalized frail older adults is currently poorly assessed and non‐demonstrated.^17^ In our study, we did not conduct a simple intervention. CGA addresses a holistic management plan for older people who could involve the multidisciplinary team full spectrum. Our results show improvements in frailty outcome when a geriatrician care plan is followed. We acknowledge that this second ‘per protocol’ analysis could introduce some selection bias, but we also think it may reflect more accurately the real effect of the treatment when applied in daily clinical practice. In addition, this finding reinforces the need of a direct involvement of a geriatric team in the implementation of the recommendations.

To date, dozens of tools have been used to capture the frailty syndrome,^27^ with different grades of feasibility, administration time, concordance and different administration times between them.^24^ This fact suggests that different frailty tools capture different frailty constructs with different sensitivity and specificity depending on the setting in which the tool is administered, and the adverse event which clinicians or researchers want to predict.^25^ According to the current results, the intervention had a beneficial effect in terms of mortality no matter the tool used to assess frailty, but this fact was not seen in the case of the functional decline as assessed through the Barthel index. Only frail participants according to the FP shown an improvement after intervention. This fact seems to confirm the hypothesis of the existence of different frailty constructs with different clinical and subclinical factors underlying or expressing this syndrome, with divergent evolutions and where disparate treatments could be considered.

The number of those admitted to care home was lower than forecasted, and did not allow us to run any type of analysis and, accordingly, of reaching any firm conclusion about this outcome.

We must mention some limitations of our study. The intervention was implemented according to the means and professionals involved in every site and the local clinical pathways available in each one of them. In addition, we have not directly approached the issue of differences between countries, which was not among the purposes of the study and for which we did not have enough statistical power. However, we must take into account that the intervention was done added, but not instead of, to the usual care provided in every hospital. This fact supports that the benefits are accounted by the intervention, although differences in local practices cannot be absolutely excluded as a potential explanation for our findings, making sensible to replicate the study in countries with different organization of their health and social services.

Moreover, co‐morbidity was higher in the IG than in the CG at baseline, a fact that could interfere with the results. To tackle with this potential source of bias, we have adjusted by co‐morbidity in the analysis and, in any case, the bias should be in the opposite address, as the group with the highest co‐morbidity (and potentially poorer results) is the intervention one. Another limitation that should be mentioned is the lack of information about functional status of the participants at discharge of the participants. Taking into account that the intervention started during hospitalization and that some studies have shown that indeed short‐term interventions during hospitalization can produce benefits on functional status,^28^ we cannot exclude that patients in the intervention group could show some benefit at the time of discharge. Nevertheless, the study was designed to assess the effect of a comprehensive approach, embracing both in‐ and out‐hospital intervention, with the aim of assessing mid‐term benefits (3 months).

Finally, we could not perform a time‐sensitive analysis of the time to death because some logistical problems prevented to get these data from the centralized records available in one of the participating countries. However, the short time of follow‐up after discharge (3 months) makes less relevant this issue.

Our results have been obtained using two frailty tools stemming from the ‘Frailty Phenotype’ model. Studies involving individuals with frailty identified through other frailty scales based on the ‘deficit accumulation’ model of frailty would be important to be undertaken in the future.

Some contamination cannot be excluded, as the patients allocated to the CG were managed according to usual care, including the consultation with the liaison team of the Geriatric Department on demand from the treating physician. However, this contamination would lead to an underestimation of the effect, strengthening the relevance of our results.

This study has important strengths. We have enrolled a randomized controlled clinical trial with an important amount of frail older people, a total of 821 subjects from three countries, providing a high consistency to our findings. Moreover, usual high‐risk clinical settings for frail older patients were evaluated^29^ and our intervention showed to be feasible. The percentage of losses in the follow‐up is low and the assessment of the outcomes was achieved in a high percentage of the participants. Moreover, in an effort to improve the accuracy of the data and its low likelihood of bias, researchers assessing functional status at follow‐up were blind to the group to which each participant was allocated during his or her hospital stay.

## Conclusion

Comprehensive geriatric assessment performed by a geriatric team in frail older inpatients in non‐geriatric wards, both medical and surgical, and in ER, followed by a multidimensional intervention is beneficial in preventing not only functional decline at 3 months of follow‐up, but also mortality. This fact is especially true when the recommendations are followed by other specialists. These findings support the need of detecting frailty in all older patients admitted to hospital and the benefit of incorporating a comprehensive approach and treatment to improve their outcomes.

## Funding

This work was supported by grants from the European Commission Directorate General for Health and Consumer Affairs (DG SANTE) – Third Health Programme (grant agreement number), the Spanish Ministry of Economy, Industry and Competitiveness, co‐financed by the FEDER Funds (ISCIII, PI20/00977) and CIBERFES (CB16/10/00464), and MITOFUN, Fundación Francisco Soria Melguizo.

## Conflict of interest

None declared.

## Supporting information


**Data S1.** Supporting Information.

**Data S2.** Supporting Information.

**Table S1.** Clinical and functional values by setting and treatment group.
Table S2. Effect of the intervention on the secondary outcomes selecting only those who were frail according to the Frailty Phenotype or to the FRAIL scale.
Table S3. Effect of the intervention in those who were frail according to both tools (*n* = 545).
