# Supplementary material for: Effectiveness of a randomized intervention by a geriatric team in frail hospital inpatients in non‐geriatric settings: FRAILCLINIC project
Source: J Cachexia Sarcopenia Muscle. 2023 Nov 28;15(1):361–9. doi: 10.1002/jcsm.13374 (PMC10834340; doi:10.1002/jcsm.13374)
Supplement: Supplementary file 3 — Table S1. Clinical and functional values by setting and treatment group. Table S2. Effect of the intervention on the secondary outcomes selecting only those who were frail according to the Frailty Phenotype or to the FRAIL scale. Table S3. Effect of the intervention in those who were frail according to both tools (n = 545). [file JCSM-15-361-s001.docx]

## **Supplementary Tables**

Supplementary table 1. Clinical and functional values by setting and treatment group.

|  | Emergency Room | | | Cardiology | | | Elective Surgery | | | Urgent Surgery | | |
| --- | --- | --- | --- | --- | --- | --- | --- | --- | --- | --- | --- | --- |
|  | Con | Int | p-value | Con | Int | p-value | Con | Int | p-value | Con | Int | p-value |
| N (%) | 114 (28.18) | 116 (27.88) |  | 117 (28.88) | 129 (31.01) |  | 84 (20.74) | 79 (18.99) |  | 90 (22.22) | 92 (22.11) |  |
| Age (years) | 82.82 (8.62) | 84.22 (5.01) | 0.227 | 81.14 (4.03) | 81.8 (4.8) | 0.445 | 81.48 (4.**00**) | 82.19 (4.45) | 0.334 | 84.66 (5.15) | 83.83 (4.85) | 0.340 |
| Gender (% female) | 67 (58.77) | 69 (59.48) | 0.913 | 54 (46.15) | 54 (41.86) | 0.498 | 43 (51.19) | 37 (46.84) | 0.578 | 48 (53.33) | 54 (58.7) | 0.466 |
| Barthel score, mean (SD) | 79.04 (11.67) | 77.41 (12.73) | 0.445 | 86.54 (12.52) | 85.86 (12.81) | 0.795 | 87.29 (14.17) | 85.25 (13.93) | 0.267 | 87.67 (11.86) | 88.37 (11.53) | 0.712 |
| Lawton score, mean (SD) | 2.36 (1.06) | 2.7 (1.1**0**) | **0.022** | 2.44 (1.02) | 2.25 (1.06) | 0.090 | 2.13 (1.05) | 2.08 (1.1) | 0.680 | 2.41 (0.92) | 1.91 (0.82) | **0.001** |
| Charlson score, mean (SD) | 7.05 (2.35) | 7.39 (2.2**0**) | 0.261 | 6.47 (2.13) | 6.83 (2.06) | 0.150 | 6.14 (1.88) | 6.92 (2.13) | 0.029 | 6.38 (1.83) | 6.57 (2.34) | 0.924 |
| Frailty Phenotype (% Frail) | 110 (96.49) | 108 (93.1**0**) | 0.792 | 92 (78.63) | 102 (79.07) | 0.157 | 75 (89.29) | 77 (97.47) | 0.771 | 85 (94.44) | 86 (93.48) | 0.444 |
| FRAIL Scale (% Frail) | 102 (91.07) | 105 (91.3**0**) | 0.248 | 96 (83.48) | 114 (90.48) | 0.944 | 54 (65.06) | 50 (64.1) | 0.063 | 52 (57.78) | 48 (52.17) | 0.758 |
| MMSE score, mean (SD) | 23.68 (3.58) | 23.14 (3.26) | 0.277 | 25.51 (3.16) | 25.68 (3.29) | 0.531 | 25.18 (3.3) | 24.91 (3.29) | 0.582 | 23.53 (3.09) | 23.83 (2.97) | 0.551 |
| **Length of stay, mean (SD)** | **NA** | **NA** | **-** | **16.62 (12.88)** | **16.41 (12.78)** | **0.901** | **10.54 (7.72)** | **9.14 (6.20)** | **0.042** | **8.83 (10.11)** | **9.51 (11.92)** | **0.806** |
| GDS score, mean (SD) | -- | 5.51 (3.22) |  | -- | 4.94 (3.46) |  | -- | 4.34 (2.83) |  | -- | 4.63 (2.68) |  |
| MNA, mean (SD) | -- | 16.01 (5.04) |  | -- | 16.69 (5.52) |  | -- | 16.02 (4.97) |  | -- | 16.48 (4.97) |  |
| Mortality, n (%) | 11 (9.73%) | 8 (7.21%) | 0.497 | 8 (7.92%) | 7 (5.98%) | 0.573 | 5 (6.25%) | 4 (5.33%) | 0.807 | 11 (12.36%) | 8 (8.79%) | 0.436 |
| Worsening in Barthel Index, n (%) | 41 (44.09%) | 28 (29.79%) | **0.0428** | 22 (24.72%) | 24 (22.64%) | 0.734 | 23 (31.08%) | 18 (26.47%) | 0.545 | 24 (31.17%) | 30 (37.04%) | 0.437 |
| **Re-**Hospitalization, n (%) | 21 (21.00%) | 30 (28.85%) | 0.196 | 29 (30.53%) | 22 (19.64%) | 0.070 | 12 (15.19%) | 15 (21.74%) | 0.303 | 21 (25.00%) | 28 (31.46%) | 0. 346 |
| Worsening in Lawton Scale, n (%) | 30 (33.33%) | 28 (29.47%) | 0.572 | 34 (38.20%) | 29 (27.88%) | 0.128 | 34 (45.33%) | 25 (36.76%) | 0.299 | 33 (43.42%) | 47 (58.02%) | 0.0674 |
| Emergency Room, n (%) | 14 (17.07%) | 20 (24.10%) | 0.265 | 8 (10.81%) | 13 (13.54%) | 0.592 | 12 (17.65%) | 12 (19.67%) | 0.768 | 19 (30.16%) | 16 (26.23%) | 0.627 |
| Worsening in Frail Scale, n (%) | 37 (43.53%) | 34 (38.64%) | 0.513 | 36 (50.00%) | 59 (66.29%) | 0.0366 | 39 (62.90%) | 27 (44.26%) | 0.0382 | 40 (59.70%) | 37 (49.33%) | 0.216 |

In bold: p-value <0.05. GDS: Geriatrics Depression Scale. MMSE: Mini-Mental State Examination. MNA: Mini Nutritional Assessment. Values for age and all scores are shown in mean and standard deviation.

Supplementary Table S2. Effect of the intervention on the secondary outcomes selecting only those who were frail according to the Frailty Phenotype or to the FRAIL scale.

|  | Frail according to the Frailty Phenotype (n=740) | | | | Frail according to the FRAIL scale (n=626) | | | |
| --- | --- | --- | --- | --- | --- | --- | --- | --- |
|  | Intention to treat analysis | | Per protocol analysis | | Intention to treat analysis | | Per protocol analysis | |
|  | OR (95%CI) | p-value | OR (95%CI) | p-value | OR (95%CI) | p-value | OR (95%CI) | p-value |
| Re-admission |  |  |  |  |  |  |  |  |
| **All** | 0.94 (0.61; 1.46) | 0.780 | 0.75 (0.47; 1.19) | 0.217 | 1.30 (0.73; 2.31) | 0.374 | 0.77 (0.42; 1.44) | 0.414 |
| ER | 1.49 (0.68; 3.25) | 0.317 | 1.18 (0.54; 2.60) | 0.675 | 0.89 (0.33; 2.42) | 0.815 | 0.68 (0.23; 2.00) | 0.482 |
| Car | 1.06 (0.37; 3.00) | 0.919 | 0.78 (0.26; 2.32) | 0.660 | 1.90 (0.52; 6.95) | 0.333 | 0.92 (0.23; 3.64) | 0.906 |
| ES | 0.88 (0.33; 2.38) | 0.800 | 0.69 (0.23; 2.06) | 0.510 | 1.76 (0.36; 8.56) | 0.484 | 2.73 (0.46; 16.04) | 0.266 |
| US | 0.42 (0.16; 1.15) | 0.091 | 0.88 (0.33; 2.38) | 0.800 | 0.61 (0.17; 2.20) | 0.455 | 1.76 (0.36; 8.56) | 0.484 |
| Lawton Scale |  |  |  |  |  |  |  |  |
| **All** | 0.95 (0.67; 1.34) | 0.770 | 0.72 (0.50; 1.02) | 0.067 | 0.66 (0.46; 0.96) | **0.031** | 0.61 (0.41; 0.90) | **0.012** |
| ER | 0.79 (0.41; 1.55) | 0.497 | 0.87 (0.44; 1.72) | 0.681 | 0.72 (0.37; 1.41) | 0.339 | 0.85 (0.43; 1.70) | 0.651 |
| Car | 0.65 (0.32; 1.32) | 0.236 | 0.69 (0.33; 1.41) | 0.304 | 0.52 (0.26; 1.02) | 0.057 | 0.59 (0.29; 1.18) | 0.135 |
| ES | 0.73 (0.35; 1.55) | 0.415 | 0.30 (0.13; 0.69) | **0.004** | 0.38 (0.15; 0.95) | 0.039 | 0.15 (0.05; 0.45) | **0.001** |
| US | 1.35 (0.63; 2.90) | 0.436 | 0.73 (0.35; 1.55) | 0.415 | 2.20 (0.77; 6.27) | 0.140 | 0.38 (0.15; 0.95) | **0.039** |
| Emergency Room |  |  |  |  |  |  |  |  |
| **All** | 1.11 (0.69; 1.80) | 0.660 | 0.76 (0.45; 1.27) | 0.291 | 1.11 (0.68; 1.80) | 0.673 | 0.88 (0.53; 1.44) | 0.600 |
| ER | 0.62 (0.24; 1.57) | 0.311 | 0.43 (0.16; 1.15) | 0.093 | 1.44 (0.64; 3.24) | 0.383 | 1.08 (0.47; 2.49) | 0.850 |
| Car | 0.73 (0.21; 2.50) | 0.613 | 0.24 (0.05; 1.14) | 0.072 | 1.47 (0.51; 4.17) | 0.474 | 1.15 (0.40; 3.30) | 0.794 |
| ES | 1.69 (0.54; 5.31) | 0.369 | 1.85 (0.56; 6.10) | 0.312 | 0.87 (0.26; 2.93) | 0.818 | 0.78 (0.21; 2.83) | 0.704 |
| US | 1.24 (0.49; 3.15) | 0.658 | 1.69 (0.54; 5.31) | 0.369 | 0.55 (0.17; 1.84) | 0.335 | 0.87 (0.26; 2.93) | 0.818 |
| Frail Scale |  |  |  |  |  |  |  |  |
| **All** | 0.87 (0.61; 1.23) | 0.436 | 0.87 (0.61; 1.25) | 0.455 | 1.20 (0.82; 1.77) | 0.340 | 1.23 (0.83; 1.82) | 0.301 |
| ER | 0.78 (0.41; 1.49) | 0.453 | 0.73 (0.38; 1.42) | 0.356 | 0.89 (0.46; 1.73) | 0.729 | 0.89 (0.45; 1.76) | 0.736 |
| Car | 1.93 (0.95; 3.94) | 0.070 | 1.77 (0.87; 3.62) | 0.118 | 2.45 (1.22; 4.94) | **0.012** | 2.28 (1.12; 4.62) | **0.023** |
| ES | 0.51 (0.23; 1.14) | 0.101 | 0.77 (0.34; 1.76) | 0.532 | 0.64 (0.25; 1.68) | 0.366 | 0.91 (0.33; 2.53) | 0.859 |
| US | 0.47 (0.21; 1.08) | 0.074 | 0.51 (0.23; 1.14) | 0.101 | 1.28 (0.37; 4.47) | 0.700 | 0.64 (0.25; 1.68) | 0.366 |

In bold: p-value <0.05. Models adjusted by age, gender and Charlson Index. Car: Cardiology. ER: Emergency Room. ES: Elective Surgery. US: Urgent Surgery. OR: Odds Ratio. CI: Confidence Interval.

Supplementary Table S3. Effect of the intervention in those who were frail according to both tools (n=545).

|  | Intention to treat analysis | | Per protocol analysis | |
| --- | --- | --- | --- | --- |
|  | OR (95%CI) | p-value | OR (95%CI) | p-value |
| Barthel Index |  |  |  |  |
| **All** | 0.69 (0.46; 1.04) | 0.075 | 0.77 (0.50; 1.17) | 0.216 |
| ER | 0.59 (0.29; 1.18) | 0.133 | 0.70 (0.34; 1.43) | 0.331 |
| Car | 0.58 (0.24; 1.39) | 0.223 | 0.62 (0.26; 1.52) | 0.296 |
| ES | 0.67 (0.27; 1.70) | 0.400 | 0.58 (0.22; 1.56) | 0.280 |
| US | 2.52 (0.81; 7.84) | 0.110 | 0.67 (0.27; 1.70) | 0.400 |
| Mortality |  |  |  |  |
| **All** | 0.80 (0.42; 1.53) | 0.507 | 0.38 (0.18; 0.80) | **0.011** |
| ER | 0.66 (0.25; 1.77) | 0.412 | 0.32 (0.10; 1.03) | 0.056 |
| Car | 0.42 (0.09; 2.08) | 0.290 | 0.16 (0.02; 1.45) | 0.103 |
| ES | 1.69 (0.26; 10.88) | 0.579 | 0.95 (0.12; 7.65) | 0.963 |
| US | 0.74 (0.16; 3.49) | 0.702 | 1.69 (0.26; 10.88) | 0.579 |
| Re-admission |  |  |  |  |
| **All** | 1.07 (0.64; 1.76) | 0.806 | 0.85 (0.50; 1.42) | 0.528 |
| ER | 1.40 (0.62; 3.18) | 0.422 | 1.06 (0.46; 2.45) | 0.890 |
| Car | 1.12 (0.38; 3.36) | 0.835 | 0.82 (0.27; 2.54) | 0.737 |
| ES | 0.97 (0.28; 3.43) | 0.968 | 0.90 (0.24; 3.35) | 0.872 |
| US | 0.65 (0.19; 2.25) | 0.495 | 0.97 (0.28; 3.43) | 0.968 |
| Lawton Scale |  |  |  |  |
| **All** | 0.67 (0.45; 0.99) | **0.046** | 0.60 (0.39; 0.90) | **0.015** |
| ER | 0.66 (0.33; 1.33) | 0.241 | 0.77 (0.38; 1.59) | 0.481 |
| Car | 0.50 (0.23; 1.07) | 0.074 | 0.58 (0.26; 1.27) | 0.174 |
| ES | 0.42 (0.16; 1.08) | 0.072 | 0.16 (0.05; 0.49) | **0.001** |
| US | 2.36 (0.78; 7.15) | 0.130 | 0.42 (0.16; 1.08) | 0.072 |
| Emergency Room |  |  |  |  |
| **All** | 1.27 (0.69; 2.36) | 0.444 | 0.71 (0.36; 1.39) | 0.314 |
| ER | 0.73 (0.25; 2.10) | 0.559 | 0.52 (0.16; 1.67) | 0.272 |
| Car | 1.55 (0.35; 6.91) | 0.563 | 0.36 (0.06; 2.19) | 0.265 |
| ES | 3.14 (0.55; 17.78) | 0.197 | 6.06 (0.78; 47.12) | 0.085 |
| US | 0.75 (0.20; 2.78) | 0.668 | 3.14 (0.55; 17.78) | 0.197 |
| Frail Scale |  |  |  |  |
| **All** | 1.20 (0.80; 1.80) | 0.366 | 1.14 (0.75; 1.72) | 0.541 |
| ER | 0.86 (0.43; 1.71) | 0.668 | 0.79 (0.39; 1.60) | 0.513 |
| Car | 2.55 (1.18; 5.51) | **0.017** | 2.18 (1.01; 4.73) | **0.047** |
| ES | 0.76 (0.28; 2.09) | 0.599 | 1.15 (0.39; 3.35) | 0.799 |
| US | 0.94 (0.26; 3.49) | 0.931 | 0.76 (0.28; 2.09) | 0.599 |

In bold: p-value <0.05. Models adjusted by age, gender and Charlson Index. Car: Cardiology. ER: Emergency Room. ES: Elective Surgery. US: Urgent Surgery. OR: Odds Ratio. CI: Confidence Interval.
